# Challenges in Conducting Empirical Epidemiological Research with Truck and Bus Drivers in Diverse Settings in North America

**DOI:** 10.3390/ijerph191912494

**Published:** 2022-09-30

**Authors:** Susan Soccolich, Christie Ridgeway, Jessica Erin Mabry, Matthew C. Camden, Andrew Miller, Hardianto Iridiastadi, Richard J. Hanowski

**Affiliations:** 1 Division of Freight, Transit, and Heavy Vehicle Safety, Virginia Tech Transportation Institute, Blacksburg, VA 24061, USA; 2 Faculty of Industrial Technology, Institut Teknologi Bandung, Bandung 40132, Indonesia

**Keywords:** truck research, bus research, epidemiological research, driver safety, driver health

## Abstract

Over 6.5 million commercial vehicle drivers were operating a large truck or bus in the United States in 2020. This career often has high stress and long working hours, with few opportunities for physical activity. Previous research has linked these factors to adverse health conditions. Adverse health conditions affect not only the professional drivers’ wellbeing but potentially also commercial motor vehicle (CMV) operators’ safe driving ability and public safety for others sharing the roadway. The prevalence of health conditions with high impact on roadway safety in North American CMV drivers necessitates empirical epidemiological research to better understand and improve driver health. The paper presents four challenges in conducting epidemiological research with truck and bus drivers in North America and potential resolutions identified in past and current research. These challenges include (1) the correlation between driving performance, driving experience, and driver demographic factors; (2) the impact of medical treatment status on the relationship between health conditions and driver risk; (3) capturing accurate data in self-report data collection methods; and (4) reaching the CMV population for research. These challenges are common and influential in epidemiological research of this population, as drivers face severe health issues, health-related federal regulations, and the impact of vehicle operation on the safety of themselves and others using the roadways.

## 1. Introduction

In 2020, over 6.5 million commercial vehicle drivers were operating a large truck or bus in the United States [1]. Although professional driving careers are diverse in many respects, two characteristics remain consistent: professional driving jobs are often high stress and involve extended periods of low physical activity. These aspects of the job may lead to poor diet, sedentary behavior, and irregular sleep [2]. Studies have linked these characteristics to an elevated risk for many diseases and health concerns [3,4]. For example, truck and bus drivers are at least twice as likely to be obese as the general population, have higher rates of diabetes and high blood pressure, and are more likely to suffer from obstructive sleep apnea than workers from non-professional driving careers [5,6,7,8]. Over half of drivers were considered to have metabolic syndrome [4]. In addition to poor health, truck drivers are less likely than the general workforce to utilize health insurance or seek needed medical treatment [5]. Owner-operator truck drivers have been reported to have an average life expectancy 20 years shorter than the general U.S. population [9,10].

Adverse health conditions affect not only the wellbeing of professional drivers but potentially commercial motor vehicle (CMV) operators’ safe driving ability and public safety for others sharing the roadway. One study showed that drivers with three or more medical conditions had a significantly increased risk of preventable crashes [7]. Similarly, there is evidence for an association between obesity and a 50% increase in crash risk and safety-critical events [11,12]. The impact of health conditions on roadway safety in North America necessitates empirical epidemiological research to better understand and improve driver health. However, many barriers exist in this research field that influence all aspects of the research process, including study sampling, data analysis and interpretation, and valid conclusions. Although the following barriers are not unique to North America, they do not encompass all barriers facing epidemiological research in other locations. This paper identifies and discusses four challenges in conducting epidemiological research with truck and bus drivers in North America, with potential resolutions, as recognized in past and current research of this population. These challenges include (1) the correlation between driving performance, driving experience, and driver demographic factors; (2) the impact of medical treatment status on the relationship between health conditions and driver risk; (3) capturing accurate data in self-report data collection methods; and (4) reaching the CMV population for research. These challenges are common and influential in epidemiological research of this population, as drivers face severe health issues, health-related federal regulations, and the impact of vehicle operation on the safety of themselves and others using the roadways. The research team has navigated these challenges in numerous truck and bus studies over the previous decade. The intention of this review was to address issues pertaining to CMVs in North America. However, findings from previous studies and their implications may not necessarily be applicable to issues in other developing and industrialized nations. While safety issues among CMV drivers can be universal, appropriate solutions could be country-specific and culturally unique.

## 2. Challenge: Correlation between Driving Performance, Experience, and Demographic Factors

One such barrier is the complicated relationship between driver performance and driver demographic factors or personal history. Multiple factors influence driver safety performance that can interact with complex results. Thiese et al., reported that likelihood of lifetime crash involvement is associated with age, sex, and driving experience (increasing age, male sex, and increasing experience associated with increased risk) [13]. It is likely, however, that driving experience is accompanied by greater road traffic exposure and, in turn, more opportunities for crashes. Studies have also shown a link between obesity measured by body mass index (BMI) and safety-critical event risk [11,12]. Although contrasting findings have also been reported, BMI was likely colinear with other demographic or health factors associated with driver safety [13].

A recent study of over 20,000 commercial drivers highlighted the profound effect of age and driving experience on future crash risk [14]. Younger truck drivers with less experience were far more likely to be involved in safety outcomes compared to older and more experienced truck drivers. However, older drivers were more likely than their younger counterparts to have a high BMI and medical conditions that increased future crash risk. To address these issues and allow for meaningful interpretation of the data, analyses were stratified by age quartiles to examine these confounding effects separately. To better understand the interaction of age and driving experience on driver safety performance, Dunn et al., evaluated crash and violation risk in eight experience levels (ranging from 6 months or less to 30 years or more) and six age categories (ranging from 21 to 24 years old to 65 years or older) [15]. Across all age categories, low experience (less than 1 year) was associated with the highest average carrier-recorded crash rates compared to other experience levels. Drivers with low experience who were 55 years old or older had higher preventable crash rates than all other age and experience level combinations.

Age has also shown correlation with employment patterns and safety-critical event risk [16]. Using previously collected data, Camden et al., classified drivers using employment data within one of the following employment statuses: (1) continuous employment, (2) multiple employment periods, (3) ceased employment following a crash, or (4) ceased employment not following a crash [14,16]. Over 36% of drivers with ceased employment following a crash were 21 to 33 years old (compared to 20% for 34 to 43 years old, 19% for 44 to 51 years old, and 24% for 52 years old or older). Analysis of employment status and risk of crash or violation involvement was stratified by age groups. Significance and strength of the association between employment status and driver safety performance depended on driver age. Although continuous employment was most often associated with lower involvement in crashes or violations, the stratified analysis showed a reduced association for younger drivers when compared to older drivers.

The research demonstrates that within and across commercial driver studies, interactions exist between age, experience, BMI, health conditions, and driving risk. Many variables related to safety risk, employment tenure, years of experience, and age have intricate connections that require awareness during data collection, analysis, and explanation of study findings. Stratifying analyses by key variables may be helpful in identifying these relationships and understanding the implications of research findings [13,14,15,17,18]. Furthermore, given the complicated relationships between driver-related variables and driving risk, it is essential for researchers to consider the impact of unobserved heterogeneity on research conclusions.

## 3. Challenge: Impact of Medical Treatment Status on the Relationship between Health Conditions and Driver Risk

An additional challenge in epidemiological research of truck and bus drivers is understanding the impact of health condition, diagnosis, and treatment status on crash risk. The U.S. Federal Motor Carrier Safety Administration (FMCSA) requires all commercial drivers traveling interstate with a vehicle weighing over 10,000 pounds to undergo a medical exam from a licensed medical examiner [19]. Individual states may also have additional medical examination requirements for maintaining a valid commercial driver’s license. While some health conditions are disqualifying, such as Type I diabetes and epilepsy, other medical conditions require treatment for licensing, including obstructive sleep apnea (OSA) and hypertension.

OSA, a common comorbid condition among obese individuals, causes poor sleep quality and quantity, resulting in daytime sleepiness and fatigue, and can increase crash risk [20,21,22]. Recent studies reported a 7% to 8% prevalence rate of diagnosed OSA in U.S. CMV drivers, and another 6% are estimated to potentially have undiagnosed OSA as indicated from driver and medical examiner comments [14,23]. A 2010 study of bus drivers in Edinburgh, Scotland, estimated the OSA prevalence rate in bus drivers at 10% [24]. Hypertension is estimated to affect between 33% and 40% of U.S. commercial truck drivers and 45% of bus drivers, and is a risk factor for cardiovascular disease, including stroke and myocardial infarction [8,14,23,25,26]. However, treatment has a significant impact on the associated driving risk for individuals with OSA. Drivers with treated OSA or high blood pressure had comparable crash risks to drivers without these conditions; however, when these conditions were untreated, crash risk increased [14,27].

In other cases, medications for some health conditions may have adverse side effects that can affect driving safety or even be disqualifying for a driver [28,29,30]. For example, many prescription and over-the-counter medications may cause drowsiness, fatigue, or dizziness as a side effect [31]. However, there is a lack of research examining driving performance associated with the use of many medications and effective countermeasures to prevent collisions resulting from medication use [32]. This research is difficult to perform due to ethical and safety concerns for the driver and other road users; it is also difficult to predict who will experience negative side effects and control unsafe outcomes in a real-world environment. These findings support the need to assess many aspects of driver health, including medical condition information, treatment status, and medication use. Use of prescription and over-the-counter substances (including caffeine) is common in commercial truck drivers [14,31,33]. Camden et al., analyzed logbooks from 97 drivers, collected in the National Truck Driver Study [33,34]. Drivers participated in the naturalistic driving study for 4 weeks and documented daily activities and daily use of medications and caffeine. These drivers reported 9120 substance use entries over the 4 weeks; 75% of the entries were for over-the-counter and caffeine substances, and 25% were prescription medications. Further, Soccolich et al., analyzed urine samples from 202 CMV drivers for use of 84 different substances [35]. Test results showed that 17.33% of the drivers had at least one positive substance use, including prescription and over-the-counter medications.

The research illustrates that studies of truck and bus drivers cannot rely solely on driver self-disclosure of medical conditions, but also must collect data on treatment type and status. For example, drivers can be asked (a) if they are receiving medical treatment for the condition, (b) what medications, devices, or programs are included as treatment, (c) the frequency or regularity of the treatment, and (d) use of over-the-counter substances or caffeine, in case of interactions between treatment and other substance use. A valuable data collection tool for research with CMV drivers is the commercial driver Medical Examination Report, required by the DOT for all CMV drivers at least every 2 years (or more frequently for drivers with health concerns). Sections of the Medical Examination Report included open-ended comments from the driver and medical examiner regarding prior and current medical conditions, treatment for existing conditions, recommendations for future testing/specialists, and more. These data provide a wealth of information but do require time for transcription and data coding. However, these details are not captured in sections of the report requiring a binary yes/no response for diagnosed health conditions. The Medical Examination Report can be leveraged with other data sources in order to include treatment status information in analyses.

## 4. Challenge: Capturing Accurate Data in Self-Report Collection Methods

Survey research has many benefits with worker populations, including truck and bus drivers. Surveys can be administered to a large sample of drivers at specific locations (e.g., place of employment, rest stops, conventions), disseminated through management, or shared through relevant media outlets (e.g., industry newsletters, email listservs, or social media). However, researchers must understand the limitations of self-report, survey-collected data. Self-report measures are subject to biases that distort the true nature of respondents’ demographics, attitudes, beliefs, behaviors, aptitude, and health indicators, such as treatment status, medication data, or other personal health-related factors including alcohol use [36,37,38,39]. These biases represent threats to construct validity and can attenuate both variability in responses and effect sizes of results, as well as distort relationships in other untenable ways [40].

Several forms of response biases exist, but one of the most commonly identified and researched is socially desirable responding. Individuals completing what they perceive as high-stakes or sensitive assessments may respond in a socially desirable manner, in which their answers are distorted from the truth through exaggeration, downplaying, or, in some cases, are completely counterfactual [41,42]. Hickman et al., found surveyed drivers had lower mean scores compared to mean normative scores in both the Survey of Recent Life Experiences and Dula Dangerous Driving Index, a potential reflection of this limitation [14]. Strong social desirability components within their research were identified and reduced variance in driver responses, limiting significant relationships identified with safety outcomes [14].

Socially desirable responding may specifically affect research involving self-report assessments by CMV drivers as they may express possible reluctance to disclose sensitive medical data to their employer [2,7,13,23,39,43,44,45]. In a study of burnout and alcohol use in city railway transit, Cunradi et al., were concerned study participants were reluctant to share accurate information in a workplace interview on the volume of alcohol they consumed, even when confidentiality was stressed [39]. Thiese et al., discussed the probability of self-selection bias overrepresenting drivers with fewer health conditions in health-related survey studies [2,7,13,23]. Thiese at al. have also relied on data with self-reported medical conditions, the accuracy of which could be affected by socially desirable responding [2,7,13,23].

Collecting self-report, health-related data from commercial vehicle operators is further complicated by implications of certain health conditions on licensing status. During an assessment of a commercial fleet health and wellness program, drivers expressed concern that private health data collected in the program would be shared with employers and affect their employment [43]. Program staff discussed how this fear could affect the success of the health and wellness program by discouraging drivers from participating or completing health assessments truthfully. Similarly, in a study assessing a carrier OSA program, program staff reported drivers feared receiving an OSA diagnosis due to possible job termination [44]. Drivers could hide symptoms or falsify responses to OSA screening assessments to avoid diagnosis.

Not limited to individuals, the FMCSA hosts a collection of fleet-level variables as part of their compliance, safety, and accountability tracking system. Self-reported variables are collected from active fleet management, such as the number of power-unit vehicles operated and annual mileage. These numbers may be misreported due to unintentional or malicious reasons and, in turn, may influence exposure rates at the fleet level and affect subsequent analyses. Further, a chameleon carrier—that is, a fleet that reincarnates under a new name in an attempt to circumvent regulatory violations or laws—may affect fleet-level exposure rates [46].

The inconsistencies of self-report assessments should be carefully considered when developing methodologies. Researchers should take measures to limit bias or account of potential biased responses. For example, researchers could collect anonymous data or guarantee protection against data being released outside the study team. Researchers may also employ mitigation strategies during analyses. Bergen and Labonté suggested several approaches data collectors may use to minimize the impact of bias, including establishing rapport, fielding questions, or stressing accuracy on specific measures based on common socially desirable cues identified in the study [47]. The use of validated measures or assessments that can be validated during data collection is crucial for collecting authentic CMV driver responses.

Other statistical or methodological efforts to mitigate bias include adding assessments to measure social desirability itself, employing situational judgement tests to assess personality or behavioral tendencies, evaluating bias using item response theory techniques, or utilizing measures such as the Social Desirability Scale (SDS) [48,49,50]. However, a recent meta-analysis by Lanz et al., suggested the traditional SDS may provide little to no benefit in measuring either bias or a desirability trait [51]. Any effort taken to attenuate social desirability bias and other response biases should be carefully considered to match the research objectives and implications of results.

## 5. Challenge: Reaching the CMV Driver Population for Research

An additional challenge in epidemiological research of heavy vehicle drivers is difficulty in reaching and following up with participants in the long term. Reasons for this difficulty include driver and/or fleet wariness to participate in research involving data collection or tracking, high turnover in the commercial driving industry, and demanding workdays [43,44]. Many commercial drivers are wary of providing data for fear that confidential data may reach management or could be used by the federal government or law enforcement personnel. Further, carriers are reluctant to share driver information with researchers for fear that data may increase liability if personally identifying information is shared outside the research team.

Driver turnover is problematic in the trucking industry, especially in the for-hire truckload industry, which can further impede longitudinal studies or participant follow-up [52,53]. Turnover within the truckload industry often exceeds 90% annually, which results in many drivers exiting the industry or moving from carrier to carrier [52,54]. Although some driver turnover may be good (i.e., unsafe drivers leaving the industry), high driver turnover creates an unstable workforce and decreases safety. Staplin and Gish analyzed federal crash data and found a significant correlation between crash involvement and driver turnover [55]. In Camden et al., approximately 74% of drivers ceased employment without a recent crash, and the study found driver turnover to be correlated with crash involvement and experience [16]. Turnover in the bus industry has shown fewer extreme trends compared to the truckload industry, with rates as low as 10% [56]. However, similar to in studies with truck drivers, bus drivers who terminated employment had higher crash rates compared to drivers who had remained with the company [57]. Medical data collected regularly from truck drivers during their DOT-required medical exam can be a rich source of data from a large, representative sample of drivers. However, epidemiological studies using these data to assess trends at regular time intervals will not include the exact same driver set at each interval due to driver turnover or varying medical cycles [58].

Even if drivers remain with a consistent employer for the study duration, the transient and over-the-road nature of the job can make it challenging to collect repeated data. Most of a truck or bus driver’s 11- to 14-h workday is spent driving, engaged with passengers, or performing physically active job-related tasks [59,60,61,62,63,64,65]; completing additional study-related tasks or paperwork while working is challenging. Drivers’ schedules may make it difficult for them to remain in a study or to provide consistent, timely responses. Data collection methods should be developed with these job and time constraints in mind [65]. For example, data collection methods that can be performed at study recruitment or provided to the driver to submit at a later, more convenient time may have advantages over study participation recruitment followed by scheduling data collection for a future date [14,66]. Seiber et al. [5] proposed a solution to this issue by approaching drivers on-site at truck stops where they were off duty but had enough time to complete a short interview and free medical exam.

Moreover, measures that are subjective in nature (such as fatigue or sleepiness) may not necessarily be well correlated with risks of crash, and there is therefore a need to have more objective, valid, and sensitive measures [9]. Research has suggested the use of cohort studies to improve validity, although such an approach typically requires a large subject sample, effort, and expense [21,67]. As an alternative, multiple cross-sectional studies (using shorter and more focused sets of questionnaires) can be conducted, coupled with comprehensive multivariate analysis. The demographic make-up of the study sample should be compared to the population demographics to determine if a representative sample was obtained [65,68].

## 6. Conclusions: Solutions and Research Needs

The value of epidemiological research in understanding the serious health risks that truck and bus drivers face is limited by several barriers surrounding the professional driving occupation. When assessing crash risk from epidemiological data, it is important to consider the concomitant effects of age and experience on crash risk. In other words, the relationship between health status and crash risk is unclear due to the tendency for health issues and driving experience to increase with age, yet these health issues can increase crash risk and driving experience can decrease crash risk. One option may be to stratify data by age or experience to examine the effects of these variables separately. However, the most important tool when analyzing epidemiological data is to understand interactions and account for their impacts considering individual circumstances. Another barrier affecting this type of research is the lack of consistent, available, and updated health information from medical examinations. Collecting data on health status and medical diagnoses as well as treatment type and status are critical for understanding the effects of medical conditions on commercial driver risk and improving driving safety in truck and bus drivers. A possible solution to this issue is leveraging the Medical Examination Report required by the DOT for commercial vehicle drivers. The details in this report can be supplemented with research-based medical examinations. Further, researchers may leverage data owned by insurance companies to analyze safety risk. However, privacy restrictions may limit this possibility.

The nature of long-haul driving is hallmarked by over-the-road travel for days, weeks, or even months at a time, making it difficult to collect data, including surveys and repeated measurements, from these drivers. Conducting interviews or performing free medical exams at non-work sites, such as truck stops while drivers are off duty, may be a successful option for collecting data [5]. Drivers may also respond to survey questions in socially desirable ways, leading to inaccurate or biased data. Therefore, future research should focus on the development or use of valid tools to assess drivers’ medical status to collect data that will supplement self-report surveys [14,69]. A possible solution to this problem is to build trust with participants, inform participants of confidentiality methods, stress the importance of self-reporting honestly, and introduce the concept of social desirability for self-awareness. Even with these strategies in place, it remains important to find more effective and valid ways of measuring epidemiological data in truck and bus drivers. Driver monitoring devices may be used to collect health data. However, driver monitoring systems have their own challenges. One challenge is data on driver health and biometrics is protected in many countries. It may be possible to use driver monitoring devices to assess instances of safety-critical events resulting from driver health issues.

Overall, empirical epidemiological research that is representative of commercial driver populations is necessary to better understand the serious health risks that truck and bus drivers face in North America. The most important step is for researchers studying truck and bus driver populations to be aware of the numerous challenges in conducting health and safety-related research and how these challenges affect study planning, survey design, participant recruitment, data analysis, and interpretation of results. It is critical to note the challenges and solutions offered in this paper are directed toward truck and bus research in North America; epidemiological research outside of North America may require different considerations. For example, collecting survey data from drivers may be difficult in other countries, countries may limit the collection of health data as it is protected, and countries may not require or collect regular driver medical evaluations. Through awareness and acknowledgement, researchers can build valuable solutions into their study and improve epidemiological research of truck and bus drivers.

## Data Availability

Not applicable.
